# Pathway and kinetics of malachite green biodegradation by *Pseudomonas veronii*

**DOI:** 10.1038/s41598-020-61442-z

**Published:** 2020-03-11

**Authors:** Jinlong Song, Gang Han, Yani Wang, Xu Jiang, Dongxue Zhao, Miaomiao Li, Zhen Yang, Qingyun Ma, Rebecca E. Parales, Zhiyong Ruan, Yingchun Mu

**Affiliations:** 1 Key Laboratory of Control of Quality and Safety for Aquatic Products (Ministry of Agriculture and Rural Affairs), Chinese Academy of Fishery Sciences, Beijing, 100141 China; 2 Institute of Agricultural Resources and Regional Planning, CAAS, Beijing, 100081 China; 3 College of Food Science and Engineering, Bohai University, Jinzhou, 121013 China; 4 College of Bioscience and Engineering, Jiangxi Agricultural University, Nanchang, 330045 China; 5 Department of Microbiology and Molecular Genetics, College of Biological Sciences, University of California, Davis, CA 95156 United States of America

**Keywords:** Environmental microbiology, Microbiology techniques, Biotechnology, Microbiology

## Abstract

Malachite green is a common environmental pollutant that poses a great threat to non-target organisms, including humans. This study reports the characterization of a bacterial strain, *Pseudomonas veronii* JW3-6, which was isolated from a malachite green enrichment culture. This strain degraded malachite green efficiently in a wide range of temperature and pH levels. Under optimal degradation conditions (32.4 °C, pH 7.1, and inoculum amount of 2.5 × 10^7^ cfu/mL), *P. veronii* JW3-6 could degrade 93.5% of 50 mg/L malachite green within seven days. Five intermediate products from the degradation of malachite green were identified: leucomalachite green, 4-(dimethylamino) benzophenone, 4-dimethylaminophenol, benzaldehyde, and hydroquinone. We propose a possible degradation pathway based on these findings. The present study is the first to report the degradation of malachite green by *P. veronii* and the identification of hydroquinone as a metabolite in the degradation pathway.

## Introduction

Malachite green is a common triphenylmethane dye that is widely used in the textile and dyeing industries. This compound has also long been applied as a fishery medicine in China’s aquaculture industry because of its ability to control certain diseases, such as saprolegniasis^1^. However, malachite green residues in aquaculture products and the environment may threaten human health, and the potential teratogenic, carcinogenic, and mutagenic effects of malachite green have been reported since the 1970s^2,3^. Based on its hazardous properties, the Ministry of Agriculture and Rural Affairs of China banned the use of malachite green in aquaculture in 2002. Nevertheless, malachite green is still used illegally because of its low price and good sterilizing effect^4^. In addition, thousands of tons of wastewater from triphenylmethane dye production are discharged into rivers and lakes and the residues persist in soils. Triphenylmethane dye residues enter the food chain, thus considerably threatening human health^5^. Therefore, developing an efficient method for malachite green degradation is crucial.

Although triphenylmethane dyes are stable and difficult to degrade, some environmental microorganisms have evolved the ability to decolorize or degrade these dyes. These microorganisms have become the most effective “weapon” against triphenylmethane dye pollution in the environment^6,7^. Several strains of bacteria and fungi that can decolorize or degrade triphenylmethane dyes have been isolated from soils, lakes, and liquid waste. In previous studies, quite a few bacterial degradation strains such as *Aeromonas hydrophila*, *Tenacibaculum* sp. HMG1, *Pseudomonas* sp. YB2 have been reported^8–14^ (Table 1). Recently, some novel malachite green biodegradation strains have been isolated, including *Pseudomonas* sp. DY1 and *Exiguobacterium* sp. MG2, and the degradation metabolites and pathways have been studied^4,12^. Fungi that can degrade malachite green have also been isolated; these include *Cunninghamella elegans, Phanerochaete chrysosporium*, and *Irpex lacteus*^15–18^. These microbial species serve as resources for the biodecolorization of triphenylmethane dyes and the bioremediation of environmental pollution. Nevertheless, further studies are needed, as previous studies mainly isolated decolorizing bacteria through traditional cultivation methods. Thus, new and efficient methods should be explored. Moreover, only the upstream decolorization pathway of triphenylmethane dyes, such as malachite green has been reported. The complete pathway for microbial degradation and triphenylmethane dye metabolism remains unknown^19^.

**Table 1:** Reported microorganisms for degradation of triphenylmethane dyes.

Name of strain	kingdom of strain	Triphenylmethane dye type	Reference
*Aeromonas hydrophila*	Bacteria	Malachite green, crystal violet	^8^
*Arthrobacter* sp. M6	Bacteria	Malachite green	^9^
*Bacillus subtilis* sp.	Bacteria	Malachite green	^10^
*Citrobacter* sp.	Bacteria	Malachite green, crystal violet, brilliant green	^11^
*Exiguobacterium* sp. MG2	Bacteria	Malachite green, crystal violet, brilliant green	^12^
*Pseudomonas* sp. YB2	Bacteria	Malachite green	^13^
*Pseudomonas* sp. strain DY1	Bacteria	Malachite green	^4^
*Tenacibaculum* sp. HMG1	Bacteria	Malachite green	^14^
*Cunninghamella elegans*	Fungi	Malachite green	^15^
*Irpex lacteus* F17	Fungi	Malachite green, crystal violet	^16^
*Phanerochaete chrysosporium*	Fungi	Malachite green	^17^
*Pleurotus ostreatus*	Fungi	brilliant blue R	^18^

The objectives of the present study were: (1) to isolate a promising bacterial strain for the treatment of malachite green-contaminated environments; (2) to determine the kinetic parameters for the biodegradation of malachite green and other triphenylmethane dyes; and (3) to detect the metabolites and deduce the possible downstream degradation pathway of this strain, and examine the mechanism underlying malachite green degradation by *P. veronii* JW3-6.

## Results

### Diversity and community changes in enrichment cultures

**Figure 1:**
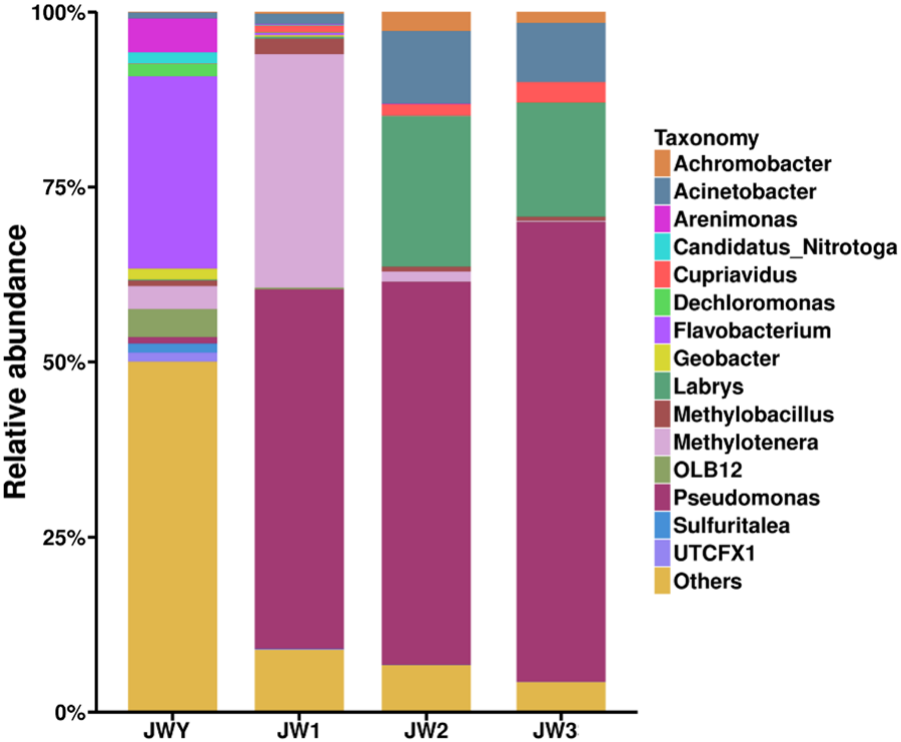
Diversity and community changes in enrichment cultures JWY, JW1, JW2 and JW3 based on the relative abundance of the Illumina sequences. The histogram was constructed by using R software.

High-throughput sequencing yielded 63,153, 80,192, 79,079, and 78,197 16 S rRNA gene sequences of the distinct V4–V5 regions of the four sequential enrichment cultures JWY, JW1, JW2, and JW3, respectively. After statistical analysis and annotation, 63,153 sequences in the initial JWY culture were clustered into 1,568 OTUs. The highest abundance of OTUs belonged to *Flavobacterium*, *Pseudomonas*, *Arenimonas*, *Methylotenera*, and *Dechloromonas* (Fig. 1). The diversity of bacteria in the first transfer culture JW1 was significantly lower, as 80,192 sequences were clustered into 967 OTUs. The main genera included *Pseudomonas*, *Methylotenera*, *Methylobacillus*, *Acinetobacter*, and *Labrys*. *Pseudomonas* replaced *Flavobacterium* as the genus with the highest abundance. The bacterial diversity after the second passage in culture JW2 also decreased, as 63,153 sequences were clustered into 576 OTUs. The main genera included *Pseudomonas, Labrys, Acinetobacter, Achromobacter*, and *Cupriavidus*. Finally, 78,197 sequences were clustered into 396 OTUs in culture JW3 after the third passage. The main microbiota belonged to *Pseudomonas*, *Labrys*, *Acinetobacter*, *Cupriavidus*, and *Achromobacter*. The above results suggest that some bacteria could not use the malachite green to grow and thus died out or were outcompeted with sequential passages of the enrichment culture. As a result, the population diversity in the microbial enrichment gradually declined with the increase of passage generations. This decline was accompanied by pronounced changes in the community composition. The dominant genus changed from *Flavobacterium* in the initial enrichment culture, to *Pseudomonas* after the first passage, and the proportion of *Pseudomonas* reached 67.8% after the third passage (Fig. 1). This result provides strong evidence that *Pseudomonas* were involved in malachite green degradation. In contrast, the rapid reduction in the proportion of *Flavobacterium* to less than 0.1% after one passage seems to indicate that *Flavobacterium* did not contribute to malachite green degradation. The increased proportion of *Labrys* in the total OTUs from 0.16% to 14.6% suggests that *Labrys* was also a major bacterial genus that participated in malachite green degradation. In addition, the relative proportions of *Acinetobacter*, *Cupriavidus*, and *Achromobacter* increased after the second passage, suggesting that these bacteria may also contribute to malachite green degradation.

### Isolation and identification of malachite green degraders

**Figure 2:**
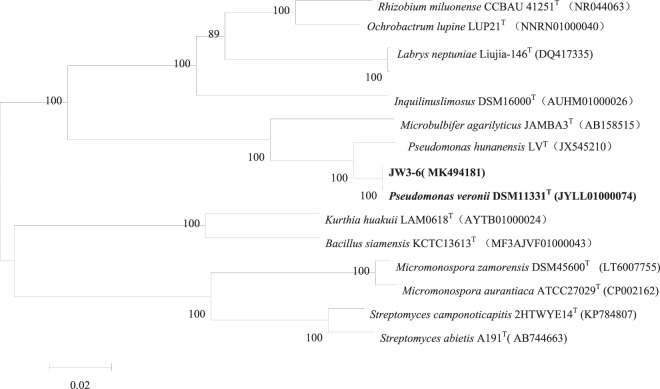
Neighbour-joining phylogenetic tree based on a comparison of the 16 S rRNA gene sequences of *P. veronii* JW3-6 and its closest relatives and several out-group strains. The numbers at the nodes indicate the percentages of bootstrap sampling derived from 1000 replications. GenBank accession numbers are given in parentheses. Bar, 0.02 nucleotide substitution per nucleotide position.

Thirteen bacterial strains able to degrade malachite green were isolated from enrichment JW3(Table 2). JW3-6 was the best malachite green degrader among the isolated strains. The degradation efficiency of JW3-6 reached 92.2%, and was significantly higher than those of other strains. JW3-6 colonies on TSA plates were milk white in color and exhibited smooth surfaces and clear edges. JW3-6 cells were Gram-negative, aerobic, nonmotile, and featured short rods that were 0.5–0.8 mm in length and 0.1 mm in width. Phylogenetic analysis indicated that strain JW3-6 belonged to the genus *Pseudomonas* and clustered strongly with *Pseudomonas veronii* DSM11331^T^ (99.69%, accession number JYLL01000074). On the basis of the results of morphological and 16 S rRNA sequence evaluation, JW3-6 was identified as a member of genus *Pseudomonas* and named as *Pseudomonas veronii* JW3-6 (Fig. 2). *Pseudomonas veronii* JW3-6 has been deposited in the China General Microbiological Culture Collection Center (CGMCC) with the accession number 17523.

**Table 2:** The taxonomic status, 16 S rRNA gene accession numbers, degradation efficiency and the growth media of the malachite green degrading strains isolated from JW3.

Name of strain	Species	Accession number of 16S rRNA gene	Degradation efficiency	Grwothmedium
JW3-6	*Pseudomonas veronii*	MK494181	91.2%	MSM
JW3-5	*Labrys neptuniae*	MN945372	59.9%	MSM
JW3-2	*Pseudomonas plecoglossicida*	MN946618	49.9%	MSM
JW3-9	*Pseudomonas umsongensis*	MN946621	28.8%	MSM
JW3-12	*Tenacibaculum mesophilum*	MN946619	23.5%	TSA
JW3-15	*Pseudomonas hunanensis*	MN946620	48.6%	TSA
JW3-18	*Rhizobium miluonense*	MN946624	35.7%	TSA
JW3-21	*Pseudomonas granadensis*	MN946622	39.2%	TSB
JW3-22	*Kurthia gibsonii*	MN946626	29.9%	TSB
JW3-34	*Inquilinus limosus*	MN946628	29.2%	LB
JW3-35	*Citrobacter sedlakii*	MN946625	35.5%	LB
JW3-39	*Pseudomonas lactis*	MN946627	25.1%	LB

### Optimization of the degradation conditions for JW3-6

**Figure 3:**
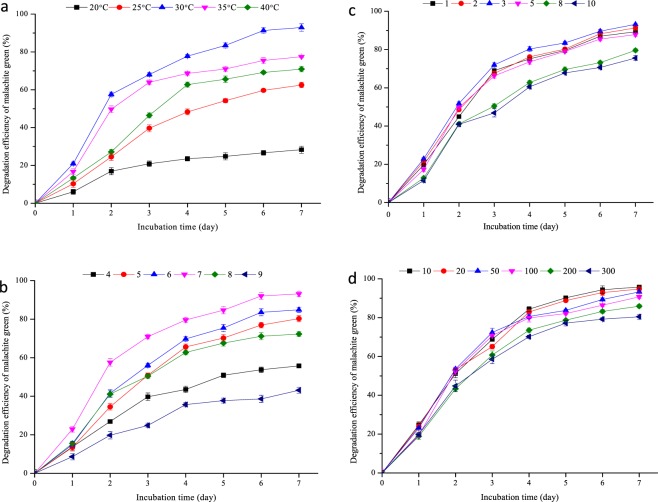
Degradation kinetics of malachite green under different conditions (**a**) temperature(°C), (**b**) pH level, (**c**) inoculum amount(×10^7^ cfu/mL), and (**d**) initial concentration(mg/L) of malachite green. The symbols represent averages of triplicate experiments and the error bars indicate their corresponding standard deviations.

The influences of multiple environmental factors on malachite green degradation by *P. veronii* JW3-6 were analyzed. This bacterial strain exhibited good malachite green degradation efficiency over a wide temperature range from 20 °C–40 °C (Fig. 3a). The degradation efficiency of *P. veronii* JW3-6 peaked (92.9%) at 30 °C and decreased to 70.1% at 40 °C. The strain can also degrade malachite green over a pH range of 4–9. The degradation efficiency of *P. veronii* JW3-6 over the pH range of 5–7 exceeded 80% and peaked (93.1%) at pH 7 (Fig. 3b). The degradation efficiency of strain under alkaline conditions was low. Degradation decreased to 55.8% when the pH was increased to 8 and further decreased to 43.1% at pH 9. This behavior indicates that *P. veronii* JW-6 exhibits good degradation under neutral or acid conditions. On the other hand, the influence of initial inoculum size on malachite green degradation by *P. veronii* JW3-6 was also studied. The degradation efficiency of *P. veronii* JW3-6 exceeded 80% when the initial inoculum was 1–5 × 10^7^ cfu/mL and peaked (92.8%) at an initial inoculum of 3 × 10^7^ cfu/mL (Fig. 3c). The initial concentration of malachite green also influenced the degradation efficiency of JW3-6. The degradation efficiency of *P. veronii* JW3-6 with 20 mg/L malachite green reached 95.1% and decreased to 81.7% when the malachite green concentration reached 100 mg/L (Fig. 3d). When the initial concentration of malachite green exceeded 200 mg/L, the degradation efficiency of *P. veronii* JW3-6 decreased to less than 70%. These results indicate that high concentrations of malachite green inhibit the growth of JW3-6.

**Figure 4:**
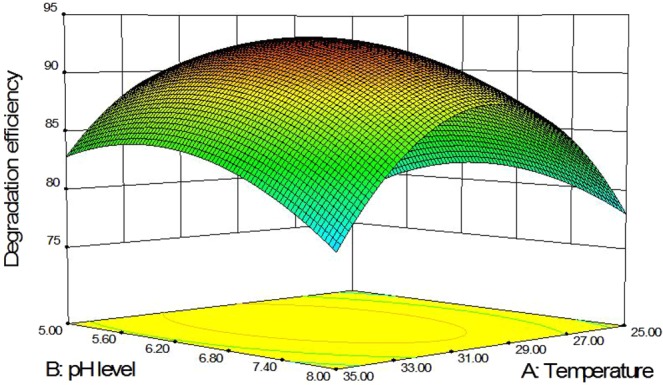
Response surface curves demonstrating the effects of medium temperature (°C) and pH level on malachite green biodegradation efficiency (%) with an inoculum amount of *P. veronii* JW3-6 at 2.5 × 10^7^cfu/mL.

Degradation conditions were further designed through the response surface method in accordance with the results of single-factor experiments, and 17 degradation tests were conducted (Table 3). The following second-degree polynomial equation was obtained through the statistical analysis of data to explain malachite green biodegradation by *P. veronii* JW3-6:

1 $${R}_{1}=93.24+0.56A\,\mbox{--}\,1.72B+0.21C-1.48AB\,\mbox{--}\,2.00AC\,\mbox{--}\,1.52BC\,\mbox{--}\,10.20{A}^{2}-3.77{B}^{2}\,\mbox{--}\,4.49{C}^{2}$$

where R_1_ denotes the predicted malachite green degradation efficiency, and A, B, and C are the coded values for temperature, pH level, and inoculum amount, respectively. Analysis of variance (ANOVA) for the fitted quadratic polynomial model is shown in Table 4. The model was significant (*P* < 0.05) with R^2^ = 0.9618 and Adj R^2^ = 0.9411. The results of regression analysis showed that temperature and pH level were significant terms (*P* < 0.05), whereas the inoculum amount was a nonsignificant term (*P* > 0.05). The response surface was used to illustrate the effects of temperature, pH level and inoculum amount on malachite green biodegradation. At the theoretical maximum point, the optimal conditions for malachite green degradation by JW3-6 were 32.4 °C and pH 7.1 and inoculum amount 2.5 × 10^7^ cfu/mL (Fig. 4).

**Table 3:** Box-Behnken experimental design with three independent variables.

Run	X_1_	X_2_	X_3_	Responses degradation efficiency(%)
1	35.0	6.5	1.0	79.8
2	30.0	8.0	5.0	80.4
3	30.0	6.5	3.0	93.6
4	35.0	8.0	3.0	75.8
5	35.0	6.5	5.0	79.7
6	25.0	6.5	1.0	73.4
7	30.0	8.0	1.0	86.5
8	25.0	8.0	3.0	78.9
9	25.0	5.0	3.0	79.8
10	30.0	6.5	3.0	93.3
11	25.0	6.5	5.0	81.3
12	30.0	6.5	3.0	93.1
13	30.0	5.0	1.0	86.5
14	30.0	6.5	3.0	92.8
15	30.0	5.0	5.0	86.5
16	35.0	5.0	3.0	82.6
17	30.0	6.5	3.0	93.4

X_1_: temperature (°C), X_2_: pH level, X_3_: inoculum amount (×10^7^ cfu/mL).

**Table 4:** Analysis of variance (ANOVA) for the fitted quadratic polynomial model.

Source	Sum of squares	DF	Mean Square	F Value	P Value
*X*_1_	2.53	1	2.53	0.63	0.4532
*X*_2_	23.80	1	23.80	5.93	0.0451
*X*_3_	0.36	1	0.36	0.090	0.7729
*X*_1_*X*_2_	8.70	1	8.70	2.17	0.1844
*X*_1_*X*_3_	16.00	1	16.00	3.99	0.0860
*X*_2_*X*_3_	9.30	1	9.30	2.32	0.1717
*X*_1_*X*_1_	437.63	1	437.63	109.04	0.0001
*X*_2_*X*_2_	59.84	1	59.84	14.91	0.0062
*X*_3_*X*_3_	85.07	1	85.07	21.20	0.0025
Model	694.31	9	77.15	19.22	0.0004
Error	0.37	4	0.093		
Total	722.40	16			

*P Value* < 0.05 indicates the model terms are significant.

### Degradation of various triphenylmethane dyes

**Figure 5:**
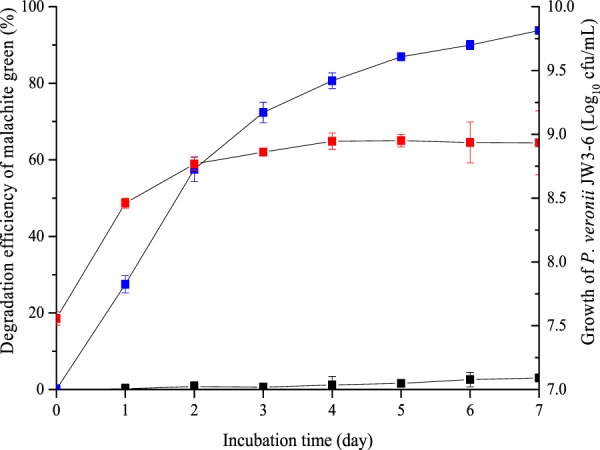
Growth of *P. veronii* JW3-6 along time and degradation efficiency of malachite green. Symbol: blue square, malachite green degradation by *P. veronii* JW3-6; black square, malachite green degradation by killed *P. veronii* JW3-6 cells as negative control; red square, the cell growth of *P. veronii* JW3-6. Data represent mean values of three replicates with standard deviation.

**Figure 6:**
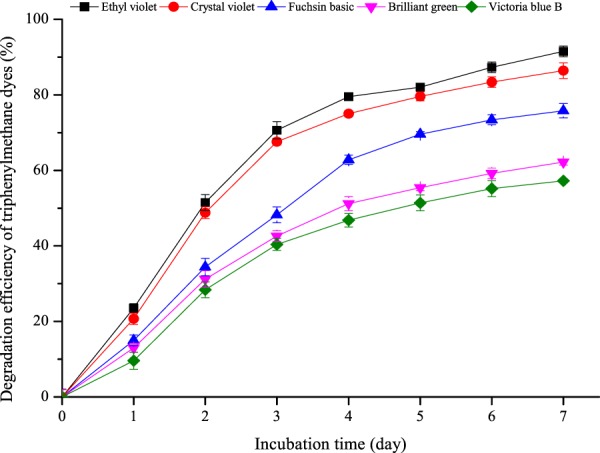
Degradation kinetics of various triphenylmethane dyes by *P. veronii* JW3-6. The symbols represent averages of triplicate experiments and the error bars indicate their corresponding standard deviations.

The degradation of malachite green and triphenylmethane dyes by *P. veronii* JW3-6 were studied under the optimal conditions. The results showed that *P. veronii* JW3-6 degraded most of the malachite green within the first 4 days, and its degradation efficiency exceeded 80% on day 4 (Fig. 5). Meanwhile, the cell density of strain JW3-6 increased to approximately 8.92 × 10^8^ cfu/mL during this period of malachite green degradation. The degradation efficiency decreased after 4 days, and was accompanied by no further increase in JW3-6 cell density. Under the optimal conditions, the degradation efficiency of *P. veronii* JW3-6 reached 93.5% on day 7 when the initial concentration of malachite green was 50 mg/L. *P. veronii* JW3-6 was also capable of degrading other triphenylmethane dyes, most likely because of their similar chemical structures. The degradation efficiencies of strain JW3-6 for ethyl violet, crystal violet, fuchsin basic, brilliant green, and victoria blue B were 91.5%, 86.4%, 75.8%, 62.2%, and 57.2% (Fig. 6). These results show that *P. veronii* JW3-6 has a broad specificity for the degradation of triphenylmethane dyes and has considerable potential for processing triphenylmethane dye pollution in the environment.

### Metabolic pathways of malachite green degradation by P. veronii JW3-6

**Figure 7:**
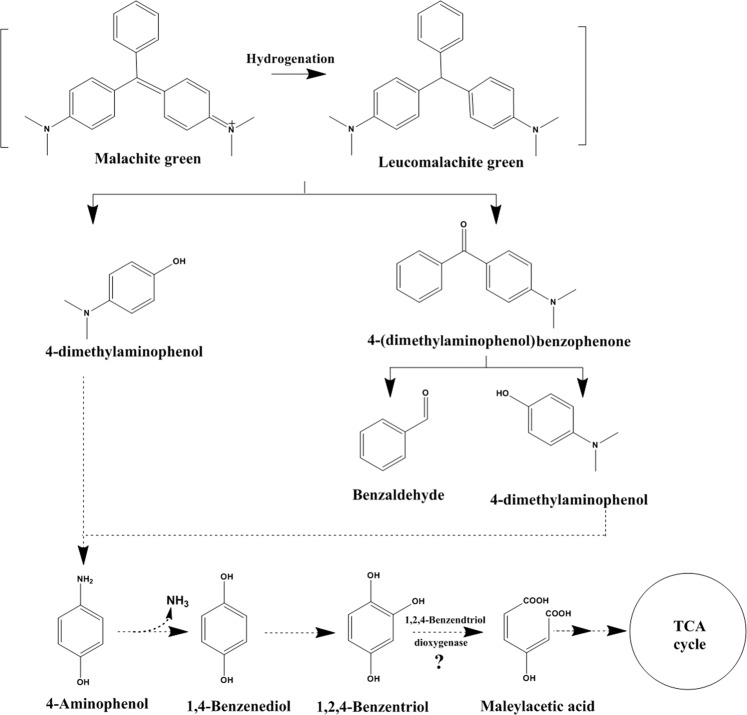
Proposed pathway for the degradation of malachite green by *P. veronii* JW3-6.

LC–MS detected five intermediates of malachite green degradation by *P. veronii* JW3-6. The candidate products were identified in accordance with their m/z values. These products were leucomalachite green (330 m/z), 4-(dimethylamino) benzophenone, 4-dimethylaminophenol, benzaldehyde, and hydroquinone. A possible metabolic pathway for malachite green biodegradation by *P. veronii* JW3-6 was proposed in accordance with the chemical properties of malachite green and the metabolites (Fig. 7). In this proposed pathway, malachite green is first transformed into leucomalachite green through the hydrogenation of the central carbon. Then, leucomalachite green is degraded via dibenzocyclization into two metabolites: 4-(dimethylamino) benzophenone and 4-dimethylamino aminophenol. These metabolites were further degraded by JW3-6; 4-(dimethylamino) benzophenone was decomposed into 4-diaminophenol and benzaldehyde through C–C bond cleavage, and 4-diaminophenol might be further degraded into hydroquinone by the action of esterases and deamination. Although the ring-opening products of hydroquinone were not detected in the present study, hydroquinone could be further degraded by phenyldiphenyl-degrading microorganisms until CO_2_ and water are produced.

## Discussion

*P. veronii* JW3-6 was isolated from an enrichment culture by means of culture dependent techniques and also retrieved by high-throughput sequencing methods. The strain is highly effective in degrading malachite green and other triphenylmethane dyes. *P. veronii* is well known for its ability as a biological control agent; it exhibits antagonistic activity against nematodes of agronomic importance^20^. However, the use of *P. veronii* in bioremediation of environmental pollutants has received inadequate attention. Several reported species of *Pseudomonas* can degrade xenobiotic compounds, such as sulfonylurea herbicides and pyrethroid pesticides^21,22^. Thus far, however, the biodegradation of malachite green and other triphenylmethane dyes by *P. veronii* strains has not been reported. This study is the first to report the isolation of a *P. veronii* strain that efficiently degrades a wide range of triphenylmethane dyes.

In the present study, *P. veronii* JW3-6 utilized malachite green as the sole carbon source, and it could degrade 93.5% of 50 mg/L malachite green within seven days. Although several malachite green-degrading bacterial strains have been isolated, most of them degrade malachite green in a nutrient-rich medium or require an additional carbon or nitrogen source in the medium. For example, Qu *et al*.^13^ reported that *Tenacibaculum* spp. HMG1 can decolorize 98.8% of 20 mg/L malachite green in 12 h in LB medium. Tao *et al*.^14^ reported that *Pseudomonas* spp. YB2 can almost completely decolorize 1000 mg/L malachite green within 12 h in LB medium. However, achieving similar degradation efficiencies in the natural environment presents difficulties. Another important property of *P. veronii* JW3-6 is that it can degrade malachite green and other triphenylmethane dyes over a wide range of temperatures (20 °C–40 °C) and pH levels (4–9). These results demonstrate the potential of this microorganism for use in biodegradation and open up new opportunities for its future applications.

Several bacteria and fungi have been isolated from various environmental samples, including soils, lakes, and liquid waste, and most of these studies focused on malachite green decolorization. However, some of the decolorization products of malachite green, such as leucomalachite green, are more toxic than the dye itself. Therefore, studying the metabolites and complete catabolic pathways of microorganisms that degrade malachite green is important. According to existing studies, different bacteria might have similar pathways of triphenylmethane dye decolorization. Ioth *et al*. identified that Michler’s ketone and diaminophenol are the main metabolites of crystal violet degradation by *Bacillus subtilis* IF0 13719 and *N. coralline*^10^. Wang *et al*. detected six metabolites from malachite green degradation by *Exiguobacterium* sp. MG2 and proposed a possible metabolic pathway^12^. In this pathway, malachite green produces leucomalachite through hydrogenation. Then, leucomalachite is cleaved into (4-dimethylamino-phenyl)-phenyl-methano through the de-benzene ring reaction. Subsequently, (4-dimethylamino-phenyl)-phenyl-methano is transformed into 3-dimethylamino-phenol and benzaldehyde through further cleavage of C–C bonds. The upstream metabolites and metabolic pathways identified in the present study are similar to those identified in previous works. Although ring-opening products were absent, 4-dimethylaminobenzene and hydroquinone were detected as metabolites. Hydroquinone could be transformed into 1,2,4-benzentriol through catalysis by phenol hydroxylase, which has been purified from certain bacteria, such as *Pseudomonas stutzeri* OX1^23,24^. In addition, 1,2,4-benzentriol can be converted into maleylacetate through catalysis by 1,2 dioxygenase. Then, the further degradation of maleylacetate produces CO_2_ and water. A new possible degradation pathway is proposed for the microbial degradation of malachite green.

In previous studies, malachite green degrading bacteria were mainly isolated through traditional methods, and the direct enrichment and isolation of degradative microorganisms were implemented. In this study, samples were first subjected to high-throughput sequencing to identify the primary members of the microbial community. Microbial changes during the enrichment of different generations of malachite green-degrading bacteria were analyzed to identify possible strains involved in malachite green degradation. The increased proportions of *Pseudomonas* and *Labrys* in from 9.39% to 67.8% and from 0.16% to 14.6%, respectively, after continuous enrichment passage indicate that these strains are likely to participate in malachite green degradation. Subsequently, 13 strains, including *P. veronii* and *Labrys neptuniae*, with malachite green degrading ability were isolated on different media, such as MSM, LB, TSA and TSB. Among them, *P. veronii* JW3-6 was found to be capable of utilizing malachite green and other triphenylmethane dyes as the sole carbon source for growth over a wide range of temperature and pH levels. On the other hand, microbial composition could be characterized through high-throughput sequencing. Moreover, the bacterial genera involved in malachite green degradation can be predicted from these data and then specific strains can try to be isolated and screened for the desired degradation ability. Overall, this work also provides a combined strategy for the isolation of microorganisms in the degradation of other environmental pollutants.

## Methods

### Chemicals and media

Malachite green (analytical grade, >98.5%) was purchased from Aladdin Industrial Shanghai Co., Ltd., China. Analytical-grade acetonitrile was purchased from Sigma–Aldrich, USA. All other reagents used were of analytical grade. The microbial community was enriched using modified mineral salt medium (MSM) modified with glucose (1.0 g/L) and supplemented with 20 µL trace element solution^25^. LB (Difco), tryptic soy agar medium (TSA, Difco), and tryptic soy broth (TSB, Difco) were used to isolate degradative strains.

### Microbial consortium construction and high-throughput sequencing

Activated sludge was collected from a purification tank for liquid waste in a dyestuff factory in Hangzhou City, Zhejiang Province, China. Samples were collected in sterile bottles and then transported back to the laboratory for subsequent tests. In the laboratory, 5 g of activated sludge was added to a 250 mL triangular flask with 100 mL MSM supplemented with 100 mg/L malachite green as the sole carbon source. The culture was then incubated on a rotary shaker (150 rpm) at 30 °C in the dark. After incubation for 7 days, portions of the culture (10%, v/v) were transferred to fresh MSM supplemented with 200 mg/L malachite green and incubated for another 7 days. A third passage was carried out in MSM containing 300 mg/L malachite green^26^. The sludge sample was designated as JWY, and the three generations of enrichment cultures were designated as JW1, JW2, and JW3. Total DNA from each of the samples was extracted by using a total soil DNA kit (Omega, USA). The 16 S rRNA genes of distinct regions V4–V5 were amplified with the following primers: 515 F (5′-GTGCCAGCMGCCGCGGTAA-3′) and 806 R (5′-GGACTACHVGGGTWTCTAAT-3′)^27^. After the purification of polymerase chain reaction (PCR) products, a library was constructed by utilizing the DNA Library Prep Kit for Illumina (NEB, USA). The amplicon library was submitted to the Maggi Technologies Company for PE2500 sequencing. Sequence analyses were performed by using Arch software (V10, http://www.drive5.com/usearch/)^28^. Sequences with ≥97% identity were assigned to the same operational taxonomic unit (OTU), and each OTU was considered to represent a species. R software(V2.15.3, https://www.R-project.org) was used for statistical analysis and histogram construction based on the relative abundance of OTUs in the samples^29^. The raw data of JWY, 1–3 is deposited at NCBI SRA database under the project PRJNA601309.

### Isolation and identification of malachite green-degrading bacteria

The JW3 enrichment culture was serially diluted and spread on LB, TSA, TSB, and MSM solid plates supplemented with 50 mg/L malachite green^12^. Bacterial colonies with different morphologies were selected and purified by repeated streaking. The malachite green-degrading ability of the isolates was detected by using a high-performance liquid chromatography (LC) system (1290, Agilent, USA) equipped with an eclipse Plus C_18_ column (4.6 mm × 150 mm, 5 µm). First, 10 mL samples were collected and an equal volume of methanol was added to each. The mixture was processed for 1 min. Then, the tube was shaken at 220 rpm for 30 min in the dark prior to centrifugation for 5 min at 5,000 rpm. The upper organic phase was collected and filtered through a 0.22 µm membrane for HPLC analysis. The elution mobile phase was comprised of a mixture of acetonitrile and distilled water (85/15, v/v) running at a flow rate of 0.6 mL/min. The injection volume was 5 µL. The photodiode array detector was operated at a wavelength of 600 nm, and the column temperature was 30 °C^30^. The analytical curve employed for malachite green quantification is shown in Figure S1. The strains with the malachite green degradation capacity were identified on the basis of their 16 S rRNA sequences. The 16 S rRNA gene fragments was amplified as described by Ruan *et al*., and purified PCR products (approximately 1.5 kb) were sequenced by Maggi Technologies Company^31^. The obtained sequences were deposited in GenBank, the accession numbers are shown in Table 2. For further identifying the strain with the highest degradation capacity, multiple sequence alignment was conducted by using Clustal X software and phylogenetic relationships were analyzed via the neighbor-joining method with MEGA 6 software^32^.

### Inoculum preparation

Strain activation was conducted before each experiment as follows. Strain JW3-6 was inoculated into MSM, incubated at 30 °C on a rotary shaker at 150 rpm in the dark. Bacterial cells in the late exponential growth phase were harvested (6 min, 8,000 rpm) at 4 °C. The supernatant was removed, and the bacterial cells were washed three times with 0.9% NaCl for subsequent studies.

### Optimization of the degradation conditions for JW3-6

The effects of temperature (20 °C, 25 °C, 30 °C, 35 °C and 40 °C), pH (4, 5, 6, 7, 8, and 9), inoculum size (1 × 10^7^, 2 × 10^7^, 3 × 10^7^, 5 × 10^7^, 8 × 10^7^, and 10 × 10^7^ cfu/mL), and initial concentration (20 mg/L, 50 mg/L, 100 mg/L, 200 mg/L, and 300 mg/L) on the degradation of malachite green by JW3-6 were investigated through a single-environmental-factor experiment. The conditions during this experiment were the same as those during activation except that the factors were selected as independent variables. To account for evaporation loss, sterile distilled water was supplemented daily according to the loss of weight.

Response surface methodology was used to further optimize the selected parameters and their interactions on the basis of Box–Behnken design^33^. The second-order polynomial equation is expressed as follows:

2 $$Yi={b}_{o}+\sum {b}_{i}Xi+\sum {b}_{ij}{X}_{i}Xj+\sum {b}_{ii}{{X}_{i}}^{2}$$

where *Y*_*i*_ refers to the predicted response, *X*_*i*_ and *X*_*j*_ are variables, *b*_*o*_ is a constant, *b*_*i*_ denotes the linear coefficient, *b*_*ii*_ represents the quadratic coefficient, and *b*_*ij*_ corresponds to the interaction coefficient.

### Biodegradation of various triphenylmethane dyes by P. veronii JW3-6

The degradation of 50 mg/L malachite green by strain JW3-6 was conducted under optimal conditions in MSM. A control was set up as described above but was inoculated with killed cells of *P. veronii* JW3-6. Each treatment was performed in triplicate. The residual malachite green concentration and cell growth were measured every 24 h. The ability of strain JW3-6 to degrade other structurally similar triphenylmethane dyes, including ethyl violet, crystal violet, fuchsin basic, brilliant green, and victoria blue B, were determined under optimal conditions. Analytical methods were the same as previously described^34^.

### Determination of malachite green biodegradation intermediates through LC–mass spectrometry (MS)

Metabolites and intermediates generated during malachite green degradation by strain JW3-6 in MSM cultures were analyzed. The extraction methods were the same as described above. Malachite green was detected by using a Xevo triple-quadrupole mass spectrometer (Waters Corp, Milford, MA, USA) equipped with an electrospray ionization source in the mass range (m/z) of 100–400. The mobile phase consisted of water and acetonitrile. The initial acetonitrile proportion was 10% and was then ramped gradually to 35% for 15 min, then to 60% for 20 min, finally to 95% for 15 min, and then held at 95% for 8 min. The injection volume was 5 µL^35^.

## Supplementary information

Supplementary information.
