# Supplementary material for: Pathway and kinetics of malachite green biodegradation by Pseudomonas veronii
Source: Sci Rep. 2020 Mar 11;10:4502. doi: 10.1038/s41598-020-61442-z (PMC7066194; doi:10.1038/s41598-020-61442-z)

**Pathway and kinetics of malachite green biodegradation by *Pseudomonas veronii***

Jinlong Song^1#^, Gang Han^1#^, Yani Wang^1^, Xu Jiang^2^, Dongxue Zhao^3^, Miaomiao Li^2,4^, Zhen Yang^1^, Qingyun Ma^2^, Rebecca E. Parales^5^, Zhiyong, Ruan^2*^, Yingchun Mu^1*^

^1^ Key Laboratory of Control of Quality and Safety for Aquatic Products (Ministry of Agriculture and Rural Affairs), Chinese Academy of Fishery Sciences, Beijing 100141, China

^2^ Institute of Agricultural Resources and Regional Planning, CAAS, Beijing 100081, China

^3^College of Food Science and Engineering, Bohai University, Jinzhou 121013, China

^4^College of Bioscience and Engineering, Jiangxi Agricultural University, Nanchang 330045, China

^5^ Department of Microbiology and Molecular Genetics, College of Biological Sciences, University of California, Davis, CA, 95156, United States of America

Correspondence to: Yingchun Mu, Zhiyong Ruan

E-mail addresses: muyc@cafs.ac.cn, ruanzhiyong@caas.cn

#Both authors contributed equally to this work.

Fig. S1 The analytical curve of HPLC employed for malachite green quantification.

Fig. S2 LC−MS spectrum of intermediates formed during the degradation of malachite green by *P. veronii* JW3-6.

a. LC−MS spectrum of leucomalachite green.


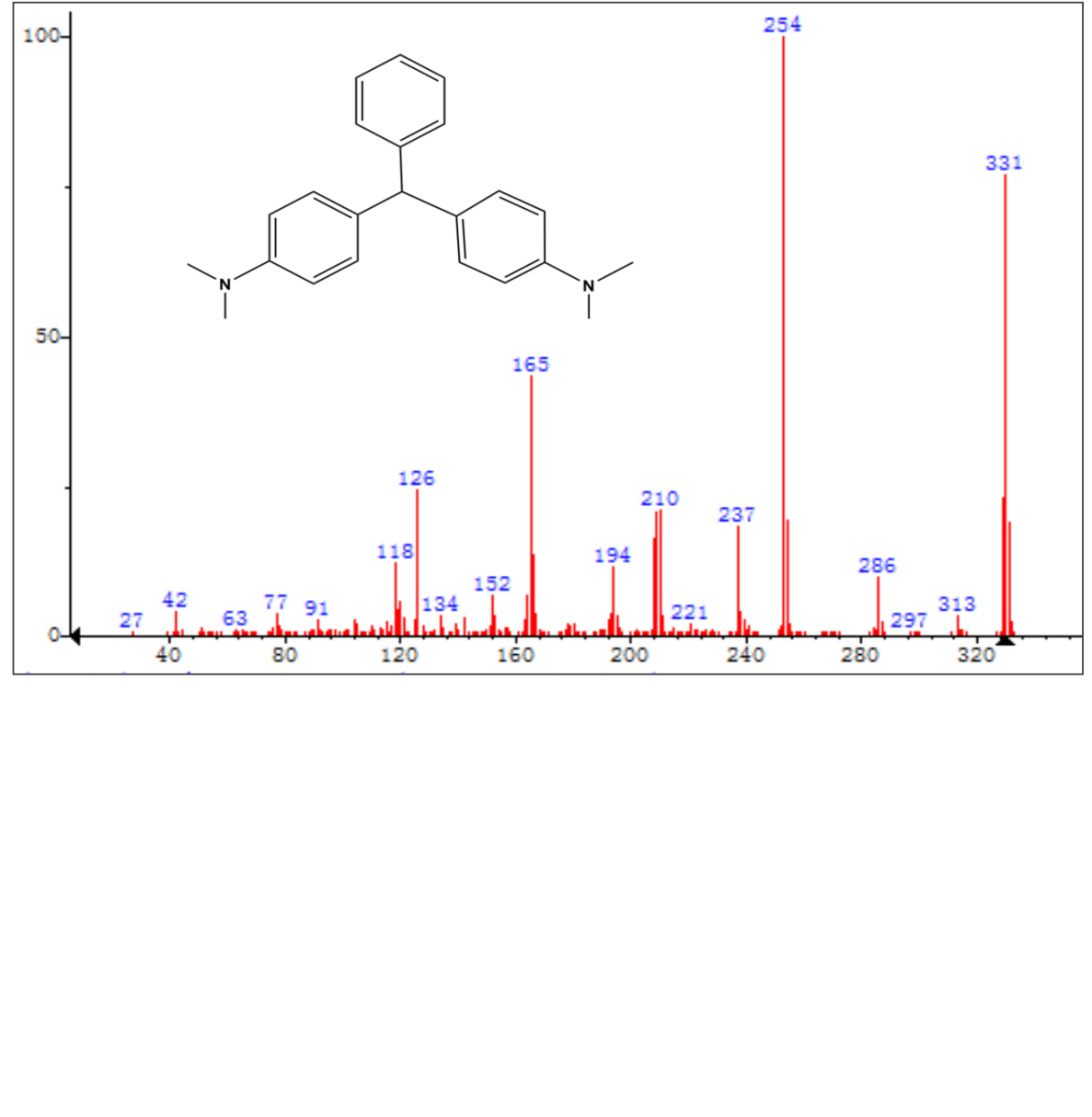


b. LC−MS spectrum of 4-(dimethylamino) benzophenone.


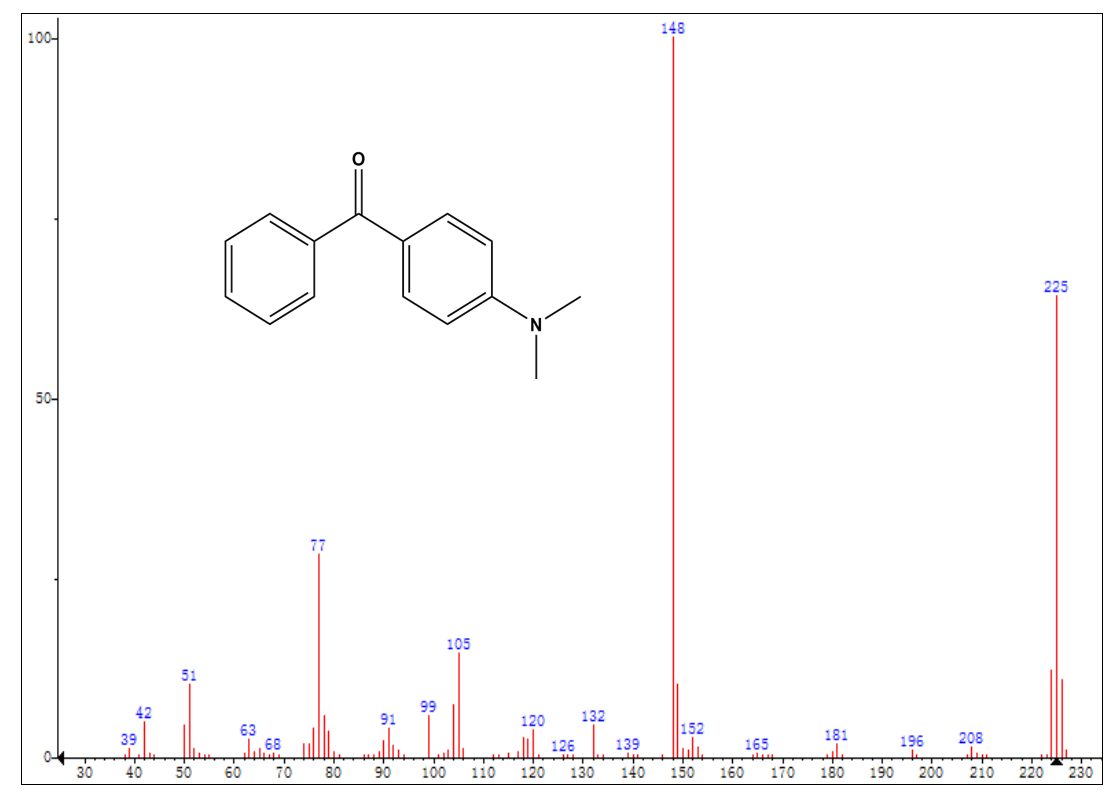


c. LC−MS spectrum of benzaldehyde.


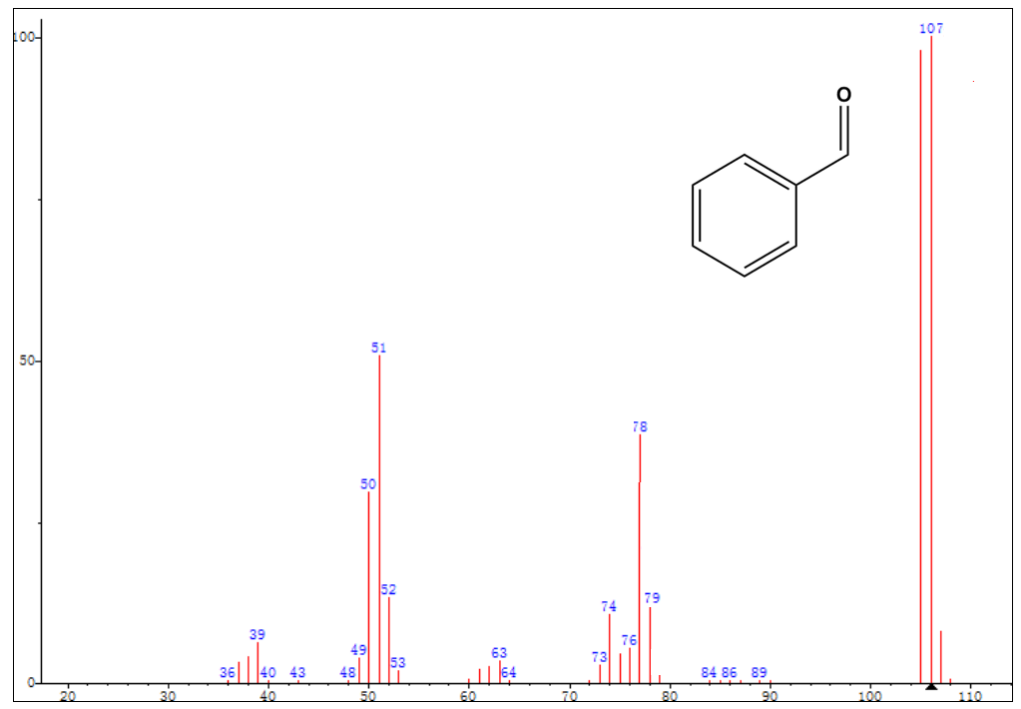


d. LC−MS spectrum of 4-dimethylaminophenol.


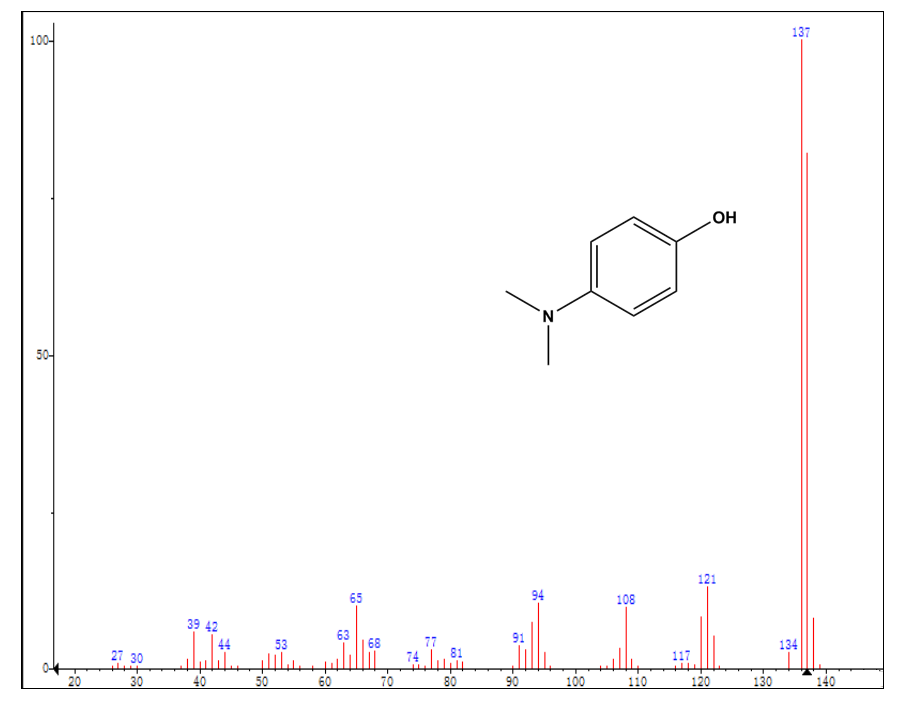


e. LC−MS spectrum of 1,4-Benzenediol.


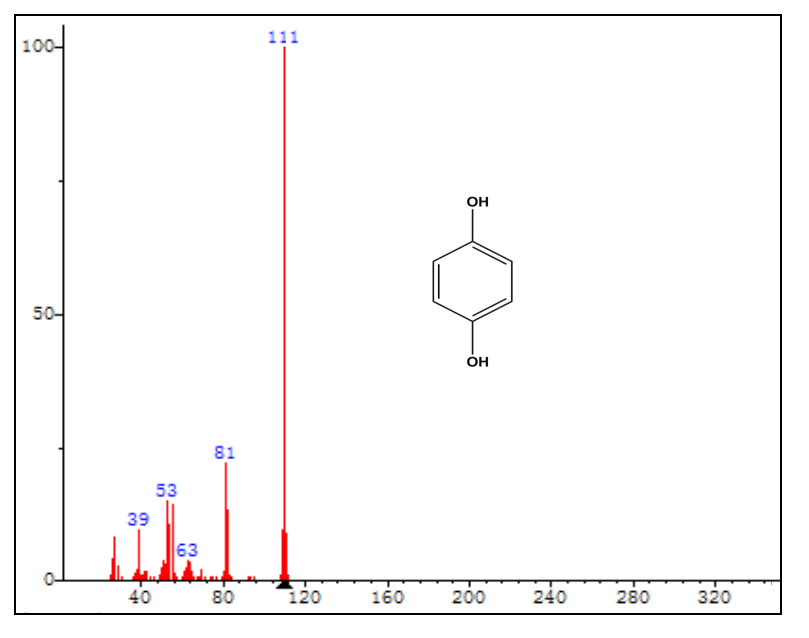

Supplement: Supplementary file 1 — Supplementary information. [file 41598_2020_61442_MOESM1_ESM.docx]
